# The Adaptive Significance of Natural Genetic Variation in the DNA Damage Response of *Drosophila melanogaster*

**DOI:** 10.1371/journal.pgen.1005869

**Published:** 2016-03-07

**Authors:** Nicolas Svetec, Julie M. Cridland, Li Zhao, David J. Begun

**Affiliations:** Department of Evolution and Ecology, University of California, Davis, Davis, California, United States of America; University of Rochester, UNITED STATES

## Abstract

Despite decades of work, our understanding of the distribution of fitness effects of segregating genetic variants in natural populations remains largely incomplete. One form of selection that can maintain genetic variation is spatially varying selection, such as that leading to latitudinal clines. While the introduction of population genomic approaches to understanding spatially varying selection has generated much excitement, little successful effort has been devoted to moving beyond genome scans for selection to experimental analysis of the relevant biology and the development of experimentally motivated hypotheses regarding the agents of selection; it remains an interesting question as to whether the vast majority of population genomic work will lead to satisfying biological insights. Here, motivated by population genomic results, we investigate how spatially varying selection in the genetic model system, *Drosophila melanogaster*, has led to genetic differences between populations in several components of the DNA damage response. UVB incidence, which is negatively correlated with latitude, is an important agent of DNA damage. We show that sensitivity of early embryos to UVB exposure is strongly correlated with latitude such that low latitude populations show much lower sensitivity to UVB. We then show that lines with lower embryo UVB sensitivity also exhibit increased capacity for repair of damaged sperm DNA by the oocyte. A comparison of the early embryo transcriptome in high and low latitude embryos provides evidence that one mechanism of adaptive DNA repair differences between populations is the greater abundance of DNA repair transcripts in the eggs of low latitude females. Finally, we use population genomic comparisons of high and low latitude samples to reveal evidence that multiple components of the DNA damage response and both coding and non-coding variation likely contribute to adaptive differences in DNA repair between populations.

## Author Summary

Understanding how genetic and phenotypic diversity are generated and maintained in natural populations is a central question in biology. Latitudinal clines in *D*. *melanogaster* represent a model system for investigating the biological and population genetic basis for local adaptation. Recent technological and statistical advances in population genomics have opened up new opportunities for investigating the extent and nature of naturally segregating variation maintained by spatially varying selection. Here, we test hypotheses generated from population genomic approaches suggesting that DNA repair is influenced by spatially varying selection in *D*. *melanogaster*. We hypothesized that UVB, which causes DNA damage and varies with latitude, could interact with genome replication stress during early embryogenesis, leading to spatially varying selection on DNA repair in this species. Here, we combine phenotypic, genetic, transcriptomic, and population genomic analyses supporting this hypothesis.

## Introduction

One of the promises of population genomic analyses is that, when combined with genome annotation, it can provide a rich source of hypotheses regarding the manifold ways in which selection may modify biological function. Because these hypotheses are relatively agnostic with regard to our preconceived notions of the traits influenced by selection and their underlying genetics, such approaches may deepen and broaden our understanding of phenotypic evolution. However, the value of these approaches is greatly enriched when population genomic-driven hypotheses regarding fitness variation can be experimentally investigated.

Population genomic analyses in *Drosophila melanogaster* have revealed that many basic cell biological processes appear to be influenced by spatially varying selection along latitudinal gradients [1–7]. How and why selection modifies these functions generally remains mysterious. For example, Turner *et al*. [5] reported that several genes associated with DNA repair harbored SNPs (single nucleotide polymorphisms) exhibiting high levels of differentiation between high and low latitude populations. Here, we extend that observation in several new directions by integrating multiple data types to produce a portrait of the diverse molecular mechanisms associated with local adaptation in DNA repair, as well as identifying a likely ecological agent of selection.

One of the main source of DNA damage in nature is the lower wavelength of solar light (Ultraviolet: UV) [8]. The sunlight UV spectrum is, by convention, divided into short (100 to 280 nm; UVC), middle (280 to 320 nm; UVB), and long wavelengths (320 to 400 nm; UVA). The UVC fraction of sunlight is completely absorbed by the higher layers of the atmosphere (stratosphere), while the UVB fraction is only partially absorbed by the lower layers of the atmosphere. Most of the solar UVA wavelengths are able to reach the earth surface. As a consequence, the latitudinal variation in solar elevation angles translates into a latitudinal cline (S1 Fig) which is steeper for UVB than for UVA [9,10]. However, due to the absorbance properties of DNA, UVB wavelengths are likely to be the main source of UV-induced DNA damage in nature [11].

UVB induces two main types of DNA lesions: CPDs (cyclobutane pyrimidine dimers) and 6-4PPs (pyrimidine-(6–4)-pyrimidone photoproducts; i.e. (6–4) photoproducts; [10,12–14]). These bulky lesions trigger multiple cellular responses aimed at detection, repair and maintenance of genome integrity. For example, a mechanism known as photoreactivation [10,15,16] relies on photolyases/glycosylases that catalyze the direct photoreversal of CPD lesions without the synthesis of new DNA. Alternatively, nucleotide excision repair generally includes the processing of several base pairs upstream and downstream of the lesion [10,17]. Failure to repair these lesions in a timely manner represents a critical threat to the cell because replicative DNA polymerases are impeded by their presence. In such cases, multiple biochemical processes are deployed to promote cell survival. These responses include the recruitment of protein complexes to stabilize stalled replications forks and the recruitment of specialized error-prone DNA polymerases able to bypass these lesions in a process known as translesion synthesis [18,19]. Moreover, DNA damage often triggers an arrest or a slowing down of the cell cycle to provide time for repair before the next cell division [20,21].

While the role of local adaptation for DNA repair as a response to geographic variation in UVB has received some attention [22–25], relatively little work [26] has been devoted to the possible influence of UVB on DNA repair variation in the genetic model system, *D*. *melanogaster*. Motivated by population genomic evidence for spatially varying selection on DNA repair proteins in *D*. *melanogaster* [5], we focused on early embryo DNA damage response as a possible target of selection for three reasons. First, as a substantial proportion of *D*. *melanogaster* eggs are laid during daytime [27], early embryos are potentially exposed to sunlight [28]. Second, the chorion transmits a significant amount of UV energy [29]. Finally, extremely rapid DNA replication during the early mitotic divisions of *Drosophila* embryogenesis leads to endogenous replication stress [21]. Thus, additional exogenous DNA damage resulting from exposure to UV during early embryogenesis could have strong fitness effects [30]. Here, we present results from phenotypic analysis, population genomics and transcriptomics supporting the hypothesis that genetic variation in the DNA damage response in *D*. *melanogaster* is maintained by spatially varying selection mediated by latitudinal variation in UVB-related DNA damage during early embryogenesis.

## Results and Discussion

### Latitudinal variation in embryo UV tolerance

**Fig 1:**
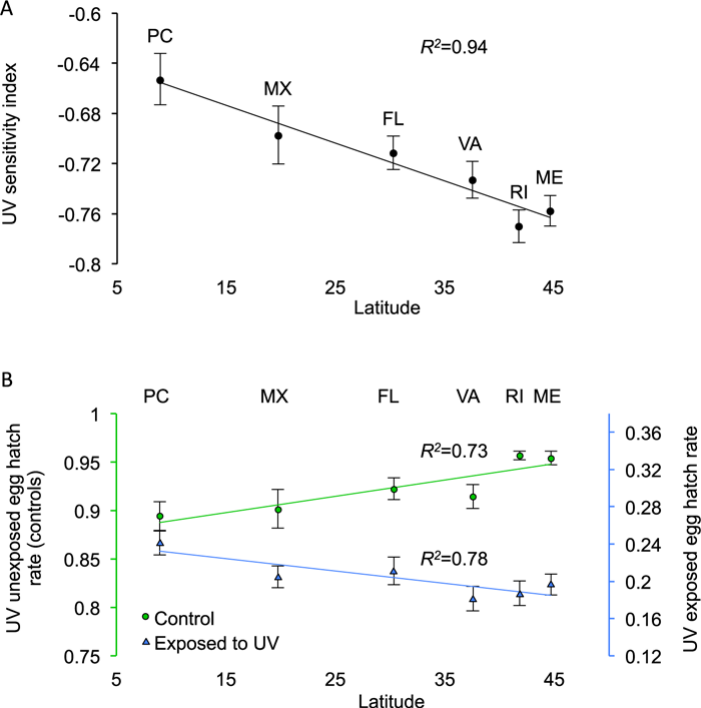
Geographic variation in UVB sensitivity among natural populations of *D*. *melanogaster* collected along a latitudinal gradient. PC (Panama, 8°N), MX (Mexico, 19°N), FL (Florida, 30°N), VA (Virginia, 37°N), RI (Rhode Island, 41°N), and ME (Maine, 44°N). We scored hatch rate for 20,328 UV-unexposed embryos (control) and for 30,853 UV-exposed embryos from 111 isofemale lines (sample sizes are: *N*_*PC*_ = 25; *N*_*MX*_ = 14; *N*_*FL*_ = 15; *N*_*VA*_ = 16; *N*_*RI*_ = 18; *N*_*ME*_ = 23). Panel (A) Regression of population mean UV sensitivity index (reduction in egg hatch rate after UV exposure) over latitude (*R*^*2*^ = 0.94, *p* = 0.001). Error bars represent the standard error of the mean (s.e.m.). Panel (B) Regression over latitude of population-mean (± s.e.m.) egg hatch rate from controls (UV-unexposed; in green; primary y-axis) and of population-mean (± s.e.m.) egg hatch rate of UV-exposed embryos (in blue; secondary y-axis). Both regressions are significant (*R*^*2*^ = 0.78, *p* = 0.019; and *R*^*2*^ = 0.73, *p* = 0.029, respectively).

We quantified geographic variation in early embryo UV sensitivity in six populations of *D*. *melanogaster* spanning 37 degrees of latitude (Fig 1A, see S2 Fig for sampling locations) for a total of 111 isofemale lines. For each line, we estimated UVB sensitivity by monitoring egg hatch rate and survival to adulthood of 1-to-3-hours old embryos unexposed to UVB (control) or exposed to a standardized dose of UVB. The population-mean embryo UVB sensitivity data strongly support the presence of a latitudinal cline (Fig 1A; linear regression: *R*^*2*^ = 0.94, *p* = 0.001; see S3 Fig for the scatterplot of UVB sensitivity *vs*. latitude for all 111 isofemale lines). The absence of geographic variation for larval-to-adult survival among the hatched individuals (linear regression: *R*^*2*^ = 0.11; *p* = 0.51) provides no support for carry-over viability effects of embryonic UV exposure on later developmental stage. The observed population differentiation in embryo UVB sensitivity corresponds to a 3.1% difference in egg hatch rate for every 10 degrees of latitude, which is comparable to previously observed clines in *D*. *melanogaster* for phenotypes such as body size and thermotolerance [31–34].

One hypothesis to explain the maintenance of fitness variation under spatially varying selection is genotype-by-environment interactions associated with trade-offs [35–38]. Regression of control hatch rates *vs*. latitude revealed a significant cline such that low latitude embryos have significantly lower hatch rates than high latitude embryos (Fig 1B; linear regression: control *R*^*2*^ = 0.78, *p* = 0.019; UV-exposed *R*^*2*^ = 0.73, *p* = 0.029). Thus, control and UV-exposed treatments both show clines, for hatch rate, though with opposite sign slopes. While this is consistent with the idea that traits associated with decreased embryo UVB sensitivity are associated with reduced embryo viability in the absence of UVB exposure, alternative explanations are possible. For example, females heterozygous for chromosome inversions on all four major chromosome arms may have reduced hatch rates due to increased rates of non-disjunction [39] and low latitude populations may be segregating many intermediate frequency inversions [39–41]. Another possibility is that lower latitude females produce lower quality eggs under laboratory conditions. To test this hypothesis we used a subset of density-controlled vials, each having 25–35 eggs from the control experiments, to estimate larval-to-adult survival for each line. Population means were obtained by averaging line means. We found a significant cline for larval-to-adult viability (*R*^*2*^ = 0.78; *p* = 0.02). While this does not rule out the possibility that reduced embryo hatch rates in lower latitude females is genetically correlated with adaptations for greater embryo DNA repair, these observations are also consistent with the hypothesis that lower latitude females produce lower quality eggs, at least under typical laboratory conditions. Importantly, regardless of the explanation for the cline for control hatch rates, these data have no bearing on the conclusion that embryo UV sensitivity varies clinally.

### DNA-repair capacity assay: Oocyte repair of mutagenized sperm

**Fig 2:**
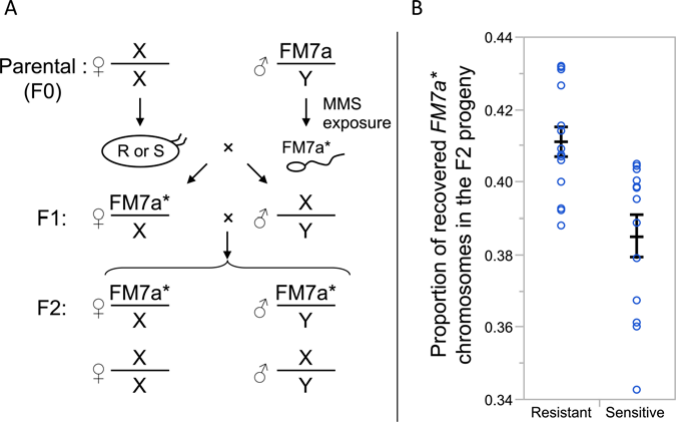
DNA-repair capacity assay: Oocyte repair of mutagenized sperm. (A) Crossing scheme of the experiment. Only the genotype of the first pair of chromosomes (*X/Y*) is shown. Parental females (F0) from either UV resistant or sensitive lines (*i*.*e*. lines from the tails of the UV sensitivity index from the latitudinal screen) were mated to an F0 Parental male carrying an *FM7a* balancer X chromosome with *B*^*1*^ as a visual marker (Bar eyes). As those males were fed with a mutagen (MMS), they produced gametes that carried deleterious DNA lesions on the *FM7a* chromosome (FM7a*), some of which may be repaired by the oocyte cytoplasm. F1 daughters were then mated to their F1 brothers. F2 offspring were scored for sex and presence of FM7a*. (B) Estimation of DNA damage repair capacity across lines showing higher *vs*. lower embryo UVB sensitivity. The graph shows the mean proportion of recovered offspring (± s.e.m.) carrying mutagenized (FM7a*) chromosomes from a crossing scheme initiated with grandmothers (F0) from either the 14 least sensitive (*i*.*e*., most resistant) or the 13 most sensitive lines. The recovery rate was significantly greater for the less sensitive (*i*.*e*., more resistant) lines (Mann-Whitney U test: *p* = 0.0017).

Early embryo phenotypes prior to the maternal-to-zygotic transition are likely associated with genetically determined maternal effects [21]. Therefore, to investigate whether the phenotypic variation for early embryonic UVB tolerance is influenced by variation in oocyte DNA repair capacity, we took advantage of the fact that DNA repair proteins derived from maternally provided oocyte transcripts can repair damaged sperm DNA subsequent to fertilization [42,43]. Thus, we set out to determine whether lines associated with lower early embryo UVB sensitivity would be associated with higher rates of repair of chemically damaged sperm DNA. To do so we monitored the recovery of MMS- damaged *X* chromosomes (*FM7a*) in a two-generation crossing scheme (Fig 2A). While the primary types of DNA damage for UVB and MMS (methyl methansulfonate) are different (CPDs and apyrimidic sites, respectively), both types of damage may be associated with stalled replication forks and translesion synthesis [44] or double strand breaks [10,45]. More generally, alternative DNA repair pathways are likely to exhibit cross-talk and are likely to repair more than one type of DNA damage [46]. For example, nucleotide excision repair genes also contribute to MMS tolerance in *Drosophila* [47].

The mean recovery rate of the mutagenized *FM7a* chromosome in F2 descendants was 7% greater (Mann-Whitney U test: *p* = 0.0017) for parental females from embryo UVB-resistant lines *vs*. those from sensitive lines (Fig 2B). The most parsimonious explanation for these results is greater efficiency of DNA repair in oocytes from females derived from lines with lower embryo UVB sensitivity, though this experiment does not rule out the possibility that other mechanisms (such as chorion protection) may contribute to variation in embryo UVB tolerance. For the 10 Panama strains assayed for both DNA-repair capacity and embryo UVB tolerance, there is a nearly significant positive correlation between the two phenotypes (Spearman correlation: *r* = 0.612; *p* = 0.059) despite the fact that the primary types of damage induced by the two treatments are different. Thus, spatially varying selection favoring higher repair rates of UVB-mediated lesions may lead to incidentally greater repair rates for other types of lesions, such as those resulting from laboratory MMS exposure.

### Gene expression variation

We speculated that at least part of the latitudinal differences in embryo UVB tolerance might be mediated by geographic variation in maternal loading of DNA damage response mRNAs to the egg/early embryo. To investigate whether high and low latitude populations exhibit differences in early embryo transcript abundance for 211 candidate DNA damage response genes (see S1 Table for complete list), we used RNA-seq to compare the transcriptomes of 1-to-3 hour-old embryos from the Panama and Rhode Island populations. Of the 8602 genes expressed in our samples (200 of which were candidate genes), 856 (9.9%) were differentially expressed (at a false discovery rate (FDR) = 0.10; see S2 Table for the complete list). Of these 856 differentially expressed genes, 21 were DNA damage response candidates (Table 1). If DNA repair transcript abundance and repair capacity are positively correlated then differentially expressed candidate genes should tend to be more highly expressed in the Panama population, which exhibits greater embryo UVB tolerance. This prediction was strongly upheld, as 20 of the 21 differentially expressed candidates showed higher expression in Panama embryos, which represents a three-fold enrichment over transcriptome wide expectation (hypergeometric test: *p* = 2.7 × 10^−9^, assuming differential transcript abundance for each of the 21 genes is mechanistically independent).

**Table 1:** DNA repair genes differentially expressed between Panama and Rhode Island (FDR 0.1).

Gene	Human ortholog	Diff SNP with reg potential ^1^	Expression fold change^2^
*Blm*	BLM		1.25
*Brca2*	BRCA2	1	1.48
*CDC45L*	CDC45		1.19
*CG6812*	SFXN1-2	2	1.32
*Debcl*		3	1.28
*DNApol-δ*	POLD		1.36
*DNApol-ε255*	POLE	5	1.23
*DNApol-η*	POLH	1	1.38
*Gnf1*	RFC1		1.22
*grp*	CHEK1	2	1.14
*lig3*	LIG4; LIG3		1.29
*lok*	CHEK2		1.25
*mei-41*	ATR	2	1.30
*mus101*	TOPBP1		1.25
*Rad17*	RAD17	1	1.19
*rad50*	RAD50	2	1.27
*RecQ4*	RECQL4	3	1.22
*RpLP0*	RPLP0		1.36
*Snm1*	DCLRE1A-B		1.18
*Thd1*	TDG		1.15
*tos*	EXO1		1.28

^1^ SNPs located within UTRs or within 500bp upstream/downstream of UTR and also showing significant population differentiation (FDR 0.001).

^2^ Absolute PC/RI fold change in expression

While DNA damage response genes expressed in multiple tissue or developmental stages may influence several fitness components, we found that candidate genes showing differential expression between Panama and Rhode Island were 2.5 times more likely than non-differentially expressed ones to show an expression bias toward the 0-2h embryo stage in modENCODE data (likelihood ratio test: *p*< 0.001). This supports the idea that DNA damage response genes specifically affecting early embryo fitness components are the most likely targets of selection.

### Genetic differentiation associated with DNA damage response genes

To investigate the possible population genetic basis of geographic differences in embryo UVB tolerance and early embryo transcript abundance for DNA damage response genes, we estimated SNP differentiation between Panama and Maine from pooled population genomic data (See Methods). We constructed a gene-based SNP data set by identifying SNPs located between 500bp upstream of the 5’UTR and 500bp downstream of the 3’UTR for all genes in the genome. After filtering, the dataset contained 853,543 SNPs mapping to 15,599 genic regions. We then identified all genic SNPs that were differentiated between Maine and Panama populations (See M&M). At an FDR of 0.001, 95% of differentiated SNPs exhibited *F*_*ST*_ higher than 0.081 and the median *F*_*ST*_ for this set of SNPs was 0.14, corresponding to the top 5% of the whole data set *F*_*ST*_ distribution. We compared our differentiated SNPs to previously published data on *D*. *melanogaster* SNPs correlated with latitude in five populations sampled from Maine to Florida [1]. Before doing so, we re-calculated from the Bergland *et al*. [1] data, per chromosome arm *q*-values to account for the heterogeneity in genomic patterns of geographic differentiation across chromosome arms [3,4]. For the 582,149 SNPs observed in both data sets, we compared our differentiated SNPs (FDR 0.001) and their clinal SNPs (FDR 0.05) and found that 33.5% of our differentiated SNPs were identified as clinal in Bergland *et al*. [1] (hypergeometric test: *p*< 10^−10^, assuming SNP independence). Thus, as expected, alleles differentiated between Panama and Maine also exhibit correlations with latitude in independent samples.

To investigate the possible role of protein variation in embryo UVB tolerance, we searched for significantly differentiated (FDR 0.001) non-synonymous SNP (nsSNPs) in DNA damage response candidate genes. We found 122 such SNPs distributed across 61 candidate genes (for the complete list see S3 Table) and exhibiting *F*_*ST*_ values between 0.42 and 0.07. The 15 genes containing the 20 most differentiated nsSNP are listed in Table 2. A majority of these genes (8 of 15) reside on chromosome arm *3R*, and four are located in the regions spanned by *In(3R)Payne* (a polymorphic chromosome inversion that segregates in many *D*. *melanogaster* populations [41]), supporting previous results [3,4] that genomic latitudinal differentiation is strongly associated with this inversion (S4 Fig). However, among all candidate genes carrying at least one differentiated nsSNP there is no enrichment for any chromosome arm/inversion location (S5 Fig).

**Table 2:** Candidate DNA damage response genes associated with the 20 highest *F*_*ST*_ non-synonymous SNPs (FDR 0.001).

Gene name	Human ortholog	Chr. arm ^1^	Sig. nsSNPs (10^−3^ FDR) ^2^	Sig. nsSNPs (10^−5^ FDR) ^2^	Perc. tail of chr. arm *F*_*ST*_ distrib ^3^
*CG5316*	APTX	*3R*	4	2	0.5
*Claspin*	CLSPN	*3L*	13	4	1
*DNApol-ε255*	POLE	*3R*	5	2	1
*DNApol-ι*	POLI	*3R*	1	1	4.5
*Fancm*	FANCM	*3R*	5	3	4.5
*Irbp*	XRCC6	*3R*	4	2	0.5
*mh*	SPRTN	*X*	5	4	4.5
*mu2*		*3L*	1	0	1.5
*mus201*	ERCC5	*2L*	2	1	1.5
*mus205*	REV3L	*2R*	4	2	1.5
*mus308*	POLQ	*3R*	1	1	1.5
*mus312*		*3L*	4	0	2.5
*nej*	CREBBP, EP300	*X*	1	0	4
*Rbf2*	RB1, RBL1-2	*3R*	5	1	3.5
*slx1*	SLX1-4	*3R*	1	1	1.5

^1^ Chromosome arm

^2^ Significant at the given false discovery rate.

^3^ Percent tail of chromosome arm *F*_*ST*_ distribution of the most differentiated nsSNP in the gene.

To identify possible candidate-gene cis-regulatory variants influenced by spatially varying selection, we looked for differentiated SNPs (FDR 0.001) located in UTRs or 500bp upstream/downstream of UTRs. We found 260 such SNPs distributed across 108 candidate genes. Of the 21 genes differentially expressed between PC and RI, 10 contained at least one such differentiated SNP (Table 1), consistent with the idea that adaptively differentiated cis-acting variants contribute to the observed geographic differences in transcript abundance.

### Hypotheses on the genes and pathways under selection

**Fig 3:**
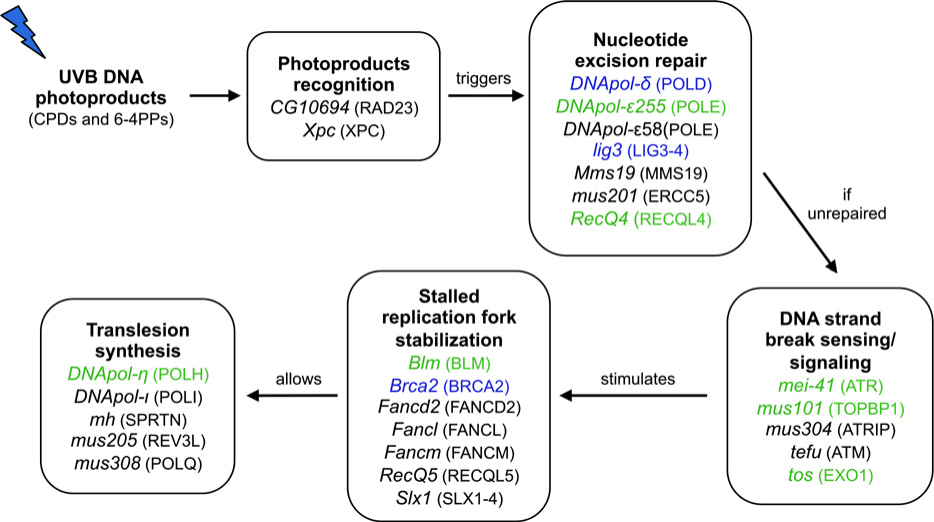
Integrating population genetic and transcriptome data into DNA damage response pathways. General pathways (bold black) and gene components (italic) and their human orthologs (in brackets) showing significant differentiation between high and low latitudes. Genes that carry at least one differentiated non-synonymous polymorphism (FDR 0.001) are shown in black; genes with early embryo differential expression between high and low latitudes in blue; genes that carried at least one differentiated nsSNP and were differentially expressed in green.

Integrating this information, we can hypothesize how selection associated with greater UVB damage at lower latitudes could affect multiple DNA damage response pathways (Fig 3; broader integration S6 Fig). Given that syncitial nuclei located at the periphery of the embryo should receive more UV energy, we speculate that most of the selection in nature occurs between mitotic cycles 8 to 14 corresponding to roughly 1.5 to 2.5 hours after oviposition [21]. Greater amounts of CPD and 6-4PPs would favor increased capacity for damage recognition and nucleotide excision repair, the repair pathway with the highest affinity for UVB photoproducts [48,49]. If nucleotide excision repair is overwhelmed, unrepaired CPDs and 6-4PPs would mobilize biochemical resources for stabilization of the resulting stalled replication forks, followed by translesion synthesis. For example, the translesion polymerase *DNApol-η*, which is especially efficient at CPD bypass [18,19] and which is known to influence UV tolerance in flies [50], shows both nsSNP and expression differentiation, supporting the idea that geographic variation in repair pathways downstream of NER may be influenced by environmental variation in UV exposure. Also noteworthy is the appearance of multiple geographically differentiated Fanconi anemia group protein coding genes. These proteins play an important role in the stabilization of stalled replication forks [51], which are a byproduct of UV-induced lesions [45,52].

Within these pathways are some examples of physically interacting proteins that appear to be influenced by spatially varying selection (Fig 3). For example, when NER is overwhelmed, *mus201 (ERCC5)* is replaced by the endonuclease *tos (EXO1)* [53,54], which results in single-stranded DNA breaks, leading to the activation of a central player in multiple DNA damage response signaling cascades, the *mei-41/mus304 (*ATR/ATRIP) dimer [55], which itself interacts with *mus101 (TOPB1)* [56]. All of these proteins show strong evidence of geographic differentiation.

Four candidate genes (*RecQ4*, *DNApol-ε255*, *DNApol-η*, and *mei-41*) are noteworthy in that they show significant geographic differentiation for i) early embryo expression differences, ii) nsSNPs, and iii) UTR or flanking SNPs. The *mei-41* gene (orthologous to the human ATR gene) is particularly unusual: it contains 3 significantly differentiated non-synonymous changes and is geographically differentially expressed. Additionally, two significantly differentiated non-coding SNPs occur in potentially regulatory sequences (BIOTIFFIN motifs 24 and 92) about 50bp upstream from the transcription start site. The MEI-41 kinase plays a central role in the DNA damage response. Once activated in response to DNA damage and replication stress, it activates by phosphorylation a large number of downstream effectors of the DNA damage response from multiple pathways [57]. It also promotes chromatin conformation changes that facilitate repair [58,59]. And finally, it signals the presence of DNA damage to the cell cycle checkpoint pathway [60,61]. MEI-41 interacts with numerous geographically differentiated proteins, including TEFU, MUS304, DNAPOL-η, CLASPIN, and MUS101 [57].

### Conclusion

While the strong latitudinal cline in UVB incidence is well known [9], the possible interaction of this variation with selection and genomic variation is not well understood. Here, we have brought together several lines of evidence in the *D*. *melanogaster* model system supporting the idea that spatially varying selection associated with UVB-mediated DNA damage during early embryogenesis maintains genetic variation in the DNA damage response.

The maintenance of genetic variation under spatially varying selection generally depends on trade-offs such that genotypes favored in some environments are disfavored in others [35,36]. Our data show that populations exhibiting lower UVB embryo sensitivity show lower embryo viability when not exposed to UVB. This is consistent with, though does not prove, that there is a viability cost associated with greater DNA repair capacity. While the existence of a trade-off between embryo DNA repair and embryo viability remains to be demonstrated, it is worth speculating on possible mechanisms. One possibility is that there is an energetic cost associated with the apparent increased maternal provisioning of DNA repair associated transcripts to the oocyte. However, given that differentially expressed DNA repair transcripts constitute only a small fraction of the early embryo transcriptome, this possibility seems unlikely. An alternative is that greater DNA repair activity is associated not only with more efficacious repair of DNA lesions, but also with unregulated interactions with the DNA leading to “repair” of undamaged nucleotides. This phenomenon, which is known as gratuitous repair [46,62] suggests a possible trade-off. An incidental effect of greater repair capacity in lower latitude genotypes could be that when UVB damage is minimal (at higher latitudes), excess repair capacity might be directed inappropriately to undamaged DNA [46]. Another possible trade-off could be that increased activity of error-prone translesion polymerases leads to the accumulation of somatic mutations during development, resulting in decreased viability. Genotype-by-temperature interactions could also play a role in the maintenance of variation in DNA damage response genes. The evidence that UVB-mediated spatially varying selection on embryo DNA damage is important in this species motivates the investigation of other phenotypes, such as female oviposition behavior [63], egg shell phenotypes, or genome size [64] that might also be influenced by such selection. Finally, several of the candidate genes mentioned here play a role in germline DNA repair processes. Thus, pleiotropic effects of spatially varying selection on somatic life history components could, in principle, influence variation in meiotic mutation or recombination in natural *Drosophila* populations.

## Materials and Methods

### Fly lines

We studied a total of six *Drosophila melanogaster* populations. Four of them originate from locations along the east coast of North America: ME in Fairfield, Maine (latitude: 44°37’N), RI in Providence, Rhode Island (41°49’N), VA in Richmond, Virginia (37°32’N), and FL in Jacksonville, Florida (30°20’N) (all sampled in September 2011). An additional population (PC) was sampled in Panama City, in Panama (8°58’N) in January 2012. A set of lines sampled from several locations in Mexico (mean latitude = 19°45’N) and obtained from Bloomington fly stock center (lines number: 14021–0231.20, 21, 22, 25–28, 30–33, 40, 41, 44) and constitute the Mexico population (MX). The sampling locations are shown on S2 Fig. The *FM7a* balancer line with the dominant *B*^*1*^ (*Bar* eyes) marker was obtained from Bloomington stock center (#785). All stocks were maintained at room temperature (23°C) on a standard yeast-cornmeal-agar food medium.

### Phenotyping UV tolerance

For each isofemale line, we generated experimental animals by allowing groups of 10 to 15 parental flies to mate and lay eggs in a vial for 3–4 days. Those vials, which contain 4ml standard food, were placed into an incubator at 25°C with 12:12 light:dark cycle and 50% humidity. The emerging offspring were anaesthetized with light CO_2_ and placed in a new empty vial containing a small plastic spoon with dyed standard fly food. The spoon was changed every 24 hours for two days for egg-laying habituation. In the morning of first collection day, a new spoon with a drop of fresh yeast-water paste was placed into the vial. 1 to 2 hours later, this first spoon was removed and replaced by another one to discard long-time retained eggs. Two hours later, groups of 35 eggs were collected with a clean needle and delicately placed on a new spoon with fresh food. All eggs were placed lying on their side on the food surface, not touching any other egg. Potentially damaged eggs or accidentally dechorionated eggs were discarded. The egg collection lasted precisely 1 hour after which the spoons with eggs were separated into two treatments groups: a control group which was left 60s on the bench and an experimental group which was immediately exposed to UV for 60s in a custom made irradiator. The irradiator consisted of a box coated with aluminum foil and built with a UVB G8T5E lamp Ushio (providing a bell shaped UV light spectrum comprised between 280nm to 350nm (peaking at 306nm) and given for 1.4W UV output at 306nm). The UVB incidence in the irradiator was 201μW/cm^2^ according to a Solartech 6.2 UVB-meter (spectral response 280-322nm peaking at 300nm). The irradiator was built with 2 fans on the top to extract the heat generated by the lamp. The temperature inside the box was monitored and did not deviated from the ambient room temperature (23°C). As the UV exposures were limited to 60s, and as the eggs were not in the fan airflow, desiccation was negligible. Immediately after exposure, all spoons were transferred to a vial with food (so that humidity remained high and to provide enough food for the larvae to develop). Vials were placed back into an incubator at 25°C with 12:12 light:dark cycle and 50% humidity. 48 hours later the spoons were, one by one, gently taken out of the vials and egg hatch was scored. Each spoon was then cautiously returned into its vial and after 16 days all vials were scored for number of adults. The egg hatch rate and number of surviving adult were calculated for each spoon (35 eggs) and averaged per isofemale line. Population averages were obtained by averaging hatch rates from lines sharing the same geographical origin. Data were then analyzed in JMP v12.0.1 (SAS institute, Cary, NC, USA)

### DNA repair capacity experiment

Virgin 4-to-5-days old males from *FM7a* balancer line were starved for 6 hours in an empty vial. Males were then transferred to a regular food vial containing a cotton ball wrapped into a kimwipe soaked with red dyed 2mM MMS in a 1% sucrose solution [42,43]. Males were transferred 24 hours later into a new regular food vial for 2 hours to recover. All males were checked for a red shiny abdomen indicating ingestion of the sucrose-MMS solution. Five mutagenized males were then placed with 10, 4-to-5-days-old parental females (F0) from different wild caught isofemale lines. Parental flies were discarded 48 hours later. F1 offspring were collected for the first 4 days of emergence of a vial. Single virgin F1 females were paired with single F1 males in a new standard food vial. These F2 vials were frozen 16 days later and F2 individuals were sexed and phenotyped for *Bar* eyes (*i*.*e*. presence or absence of mutated *FM7a*). For each line, we used a total of 20 to 30 parental F0 females. We scored sex and *Bar* in the offspring (63,600 F2 individuals total) from a total of 1060 F1 females. Two flies had *Bar* eyes of wild-type color suggesting exchange between the balancer and wild type *X* chromosome, were discarded from analysis. Across all lines, 99 F2 females that produced fewer than 25 offspring individuals were also discarded from the analysis. In the final data set the minimum number of F1 females per line was 16, and the maximum was 58 for a total of 959 females. The frequencies of the *FM7a* chromosome were then calculated per sex and per line. Data were then analyzed in JMP v12.0.

### Sample preparation for RNA-seq

We sampled embryos from each of the two populations that showed the strongest embryo UV tolerance differences (RI and PC). The embryos were sampled from a random set of 14 isofemale lines from each population. We collected 1-to-3-hours-old embryos using the same procedures as described for the embryo UV tolerance experiments. We pooled embryos from each of the 14 lines from either the RI population or from the PC population. One biological replicate thus consisted of a pool of 56 embryos (4 embryos from each of 14 isofemale lines).

Embryos were collected to prepare 3 biological replicates and were immediately transferred to Trizol for RNA extraction. Poly(A)+ RNA was prepared using an NEB mRNA isolation module (E7490S). RNA-seq libraries were constructed using NEB kits E7530S (library prep), and E7335S (Oligos). Libraries were constructed following the manufacturer instructions, except we used Aline Bioscence PCR CleanDX beads for the DNA purification steps. Individual libraries were constructed with insert size between 160–190 bp and sequenced by BGI Americas (Cambridge, MA, USA) on an Illumina Hiseq2000 platform using paired-ends chemistry and 100 cycles.

### Data analysis

In total, we generated 80.2 million cleaned paired-end reads for 6 libraries (*i*.*e*. an average of 26.7 million reads per library). Clean reads were deposited to NCBI under the SRA accession (SRP067364). Data analysis was similar to Zhao *et al*. [65]: filtered clean reads (*Q* > 20 for amino acid and *Q* > 30 for read) in each sample or replicate were aligned independently to the *D*. *melanogaster* reference genome (FlyBase 6.04) using Bowtie-based TopHat [66] program. Our experiment showed high degree of replication, with *R*^*2*^ > 0.99 for all 6 pairs of biological replicates. We used HTseq [67] to estimate read count of each gene, and then estimated differential expression using the Bioconductor package (v.2.14) in R, including DESeq2 (v.1.4.5 edgeR (version 3.0.8) and voom-limma (version 3.20.8). The Benjamini–Hochberg procedure was used to control the false discovery rate [68]. Here, we present results from DESeq2 differentially expressed genes because these results showed the greatest consistency with the other two methods. We also verified that overall expression levels were consistent across libraries and across gene classes (see S4 Table).

We calculated the development stage expression specificity of candidate genes as follows. Fastq reads from 30 development stages were obtained from modENCODE [69]. High quality reads were mapped to *D*. *melanogaster* reference genome r6.04, and uniquely mapped reads used to calculate the FPKM of each gene by Cufflinks. The development stage expression specificity (*tau*) of each gene was calculated using the same method previously used for tissue specificity [70].

### Candidate gene list construction

To construct a list of candidate genes potentially involved in early embryonic UV tolerance we pooled all the genes contained the following Gene Ontologies categories and subcategories: DNA repair complex (GO:1990391), DNA integrity checkpoint (GO:0031570), Response to UV (GO:0009411), Mitotic cell cycle checkpoint (GO:0007093), Cellular response to DNA damage stimulus (GO:0006974), Single stranded DNA binding (GO:0003697), Damaged DNA binding (GO:0003684). The gene pool was then manually curated based on the strength of support from the literature for a direct function in DNA damage response. In particular, we excluded a substantial number of genes under the cellular response to DNA damage stimulus (GO:0006974) ontology because of weak support from literature for a role in UV DNA damage response. For the same reasons we excluded the genes from the GO: mitotic spindle assembly checkpoint as well as *Tango6*, *qjt* and *Ald*. We added *Dref*, *Rbf2*, *E2f1*, *E2f2* to the list as they are transcription factors with evidence of binding in the regulatory regions of some candidates [71]. We ended up with a list of 211 genes (S1 Table).

### Genome sequencing

We generated pooled paired-end Illumina libraries (NEBNext DNA Library Prep Kit # E6040S) from flies collected from Panama City, Panama. The sequencing reads are available under the SRA accession (SRP067441). We used the Maine sequencing data from Reinhardt *et al*. [2] (Bioproject #PRJNA237820). 50 females were used in the Panama pool (daughters of wild-caught females) and 36 females (one per isofemale line) were used to generate the Maine pool. Mean sequencing coverage per pool was 77.7× for Panama and 45.1× for Maine. Reads were aligned to version 5 of the *D*. *melanogaster* reference sequence using Bowtie2 with the–-very-sensitive setting [72]. Variants were called using bcftools (samtools.github.io/bcftools) requiring a read quality score of 30 for inclusion. We required a minimum of 20× coverage at a site in both the Maine and Panama populations and at least two observations of an alternate base call between the two populations to consider it in the analysis. We also excluded all triallelic sites. Subsequent to this alignment version 6 of the *D*. *melanogaster* reference was released and we used the conversion tool on FlyBase (www.flybase.org) to update the positions in our data set to version 6 positions.

### SNP identification

We considered all positions within the range of our candidate gene list plus or minus 500bp. Within these spans we categorized synonymous and non-synonymous SNPs as well as sites that occur within introns, the 3’UTR, the 5’UTR and flanking sequence. All data used to determine this information was taken from pre-computed files on FlyBase (www.flybase.org).

### Population genetic analyses

We used two different approaches to examine differences in allele frequencies at each site. First, we generated a two by two contingency table for each site in our analysis and performed the odds ratio test for independence using the ormidp.test function in the epitools package in R (medipei.com/epitools/). This test is an exact conditional test that approximates an unconditional test, which is preferable in situations with small sample sizes. We then used the *p*-values generated by our *midp* tests to calculate the false discovery rate inherent for each chromosome arm using the bioconductor package *q*-value (http://github.com/jdstorey/qvalue).

Second, we calculated *F*_*ST*_ for each position in our data set correcting for both number of chromosomes contributing to each population pool and local coverage at that site for each pool following the method in [3]. These two measures (*q*-value of the *midp* tests and *F*_*ST*_) gave us similar results with respect to identifying significant differences between our two populations (log-linear regression: *R*^*2*^ = 0.89, *p*< 0.001).

## Supporting Information

S1 Fig Geographic variation in UVB incidence. Erythemal UV dose from 8 locations ranging between 5 and 44°N were extracted from the TEMIS satellite program (Panel A). Average yearly sums in erythemal UV doses were regressed over latitude (Panel B; Standard errors on the means were too small to be plotted on the graph). The regression shows a strong and significant linear relationship between UVB incidence and latitude (Linear regression: *R*^*2*^ = 0.98; *p* < 0.0001). Erythemal UV dose is a UV quantification derived from the erythemal irradiance, which is an integration of the UV irradiance at the ground and weighted for the wavelengths responsible for susceptibility of the Caucasian skin to sunburn (erythema). Of the global UV radiation at the ground, about 94% is UVA, 6% is UVB, whereas for the erythemal-weighted UV irradiance, 83% is UVB and 17% is UVA (Temis.nl). As a consequence, the erythemal UV dose can be considered as a suitable proxy for UVB incidence. As the satellite measures were not taking in account cloud cover, we examined unweighted UVB incidence measures taken at the ground level regardless of the weather conditions (UVMRP program; http://uvb.nrel.colostate.edu/UVB/index.jsf). We regressed UVB measures from the 12 most eastern stations (between longitude -72°W and -89°W; Panel C) along the east coast of the US. Latitudes ranged between 44°N and 33°N. We found a strong linear relationship between latitude and ground UVB incidence (Panel D; Linear regression: *R*^*2*^ = 0.93; *p* < 0.0001). Including all UVMRP stations (32) in the analysis (regardless of confounding factors like climate, altitude, or longitude), resulted in a similar regression, where latitude explained more than 55% of the UVB incidence (Linear regression: *R*^*2*^ = 0.55; *p* < 0.0001). Overall, the strong linear relationships between latitude and the yearly sums in UVB energy received at the ground level support the idea that UVB varies linearly with latitude. We chose the yearly sums data because it seems to be the best way to summarize the variation over large timescales. However we cannot exclude that the ecologically relevant UVB factors vary over shorter time scales. Unfortunately the UVMPR does not monitor equatorial latitudes but we could interpolate with relatively high confidence that the yearly sum of UVB incidence at 8°N (green dot) is about 12.2 MJ/m^2^. Thus, we estimate that ecologically relevant UVB incidence is roughly 3-fold higher at equatorial latitudes compared to temperate latitudes.
(EPS)

S2 Fig Geographic distribution of the *D*. *melanogaster* sampling sites. We studied a total of six *D*. *melanogaster* populations. Four of them originate from locations along the east coast of North America: ME in Fairfield, Maine (latitude: 44°37’N), RI in Providence, Rhode Island (41°49’N), VA in Richmond, Virginia (37°32’N), and FL in Jacksonville, Florida (30°20’N) (all sampled in September 2011). An additional population (PC) was sampled in Panama City, in Panama (8°58’N) in January 2012. A set of lines sampled from several locations in Mexico (mean latitude = 19°45’N) that were obtained from the Drosophila Species Stock Center at UCSD constituted our Mexico population sample (MX).
(PDF)

S3 Fig Regression of line mean UV sensitivity over latitude. Geographic variation in UVB sensitivity among 111 lines of *D*. *melanogaster* collected along a latitudinal gradient: PC (Panama, 8°N), MX (Mexico, 19°N), FL (Florida, 30°N), VA (Virginia, 37°N), RI (Rhode Island, 41°N), and ME (Maine; 44°N). We scored hatch rate for 20,328 UV-unexposed embryos (control) and for 30,853 UV-exposed embryos (line sample sizes are: *N*_*PC*_ = 25; *N*_*MX*_ = 14; *N*_*FL*_ = 15; *N*_*VA*_ = 16; *N*_*RI*_ = 18; *N*_*ME*_ = 23). The dots represent the mean UV index for each tested line, and the green line shows the regression of line-mean UV sensitivity index (reduction in egg hatch rate after UV exposure) over latitude (*R*^*2*^ = 0.25, *p* = 2.2 × 10^−8^).
(EPS)

S4 Fig Chromosome and inversion enrichments in candidate genes. Information on physical location of candidate DNA damage response genes from FlyBase R6.04 was used to evaluate their genomic distribution. We found that with the exception of chromosome arm *3R* (hypergeometric test: *p* = 0.006 (**); note: this *p*-value is significant after Bonferroni correction for multiple testing) none of the tested chromosome arms or regions (*2L*, *2R*, *3L*, *X*, *Y*, *Chr4*, *In(2L)t*, *In(2R)NS*, *In(3R)K*, *In(3R)Mo*, *In(3R)P*, *In(3L)P*) showed significant enrichment for UV damage response genes. This suggests that our candidate genes were slightly overrepresented on chromosome *3R*, but not within the *3R* inversions.
(PDF)

S5 Fig Chromosome and inversion enrichments in candidate genes with at least one differentiated non-synonymous SNP. UV damage response genes with at least one differentiated nsSNP (FDR 0.001) were not significantly enriched in any of the tested chromosome arms or regions (*2L*, *2R*, *3L*, *X*, *Y*, *Chr4*, *In(2L)t*, *In(2R)NS*, *In(3R)K*, *In(3R)Mo*, *In(3R)P*, *In(3L)P*). This suggests that population differentiation in our candidate genes followed the general genomic pattern.
(PDF)

S6 Fig More complete integration of population genetic and transcriptome data into DNA damage response pathways. General pathways (bold black) and gene components (italic) and their human othologs (in brackets) showing significant differentiation between high and low latitudes. Genes that carry at least one differentiated non-synonymous polymorphism (FDR 0.001) are shown in black; genes with early embryo differential expression between high and low latitudes are shown in blue; genes that carried at least one differentiated nsSNP and were differentially expressed are shown in green.
(PDF)

S1 Table List of the 211 DNA damage response candidate genes. (XLSX)

S2 Table List of 856 differentially expressed genes between Panama and Rhode Island. (XLSX)

S3 Table List of DNA damage response genes with at least one non-synonymous differentiated SNP. (XLSX)

S4 Table Early embryo transcript abundance of candidate UV damage response genes. There were no discrepancies observed between the libraries or between the different gene classes that could generate spurious false positive differential expression patterns. (^1^ Fragments Per Kilobase of transcript per Million mapped reads)
(PDF)
