# Supplementary figures and images for: The Adaptive Significance of Natural Genetic Variation in the DNA Damage Response of Drosophila melanogaster
Source: PLoS Genet. 2016 Mar 7;12(3):e1005869. doi: 10.1371/journal.pgen.1005869 (PMC4780809; doi:10.1371/journal.pgen.1005869)

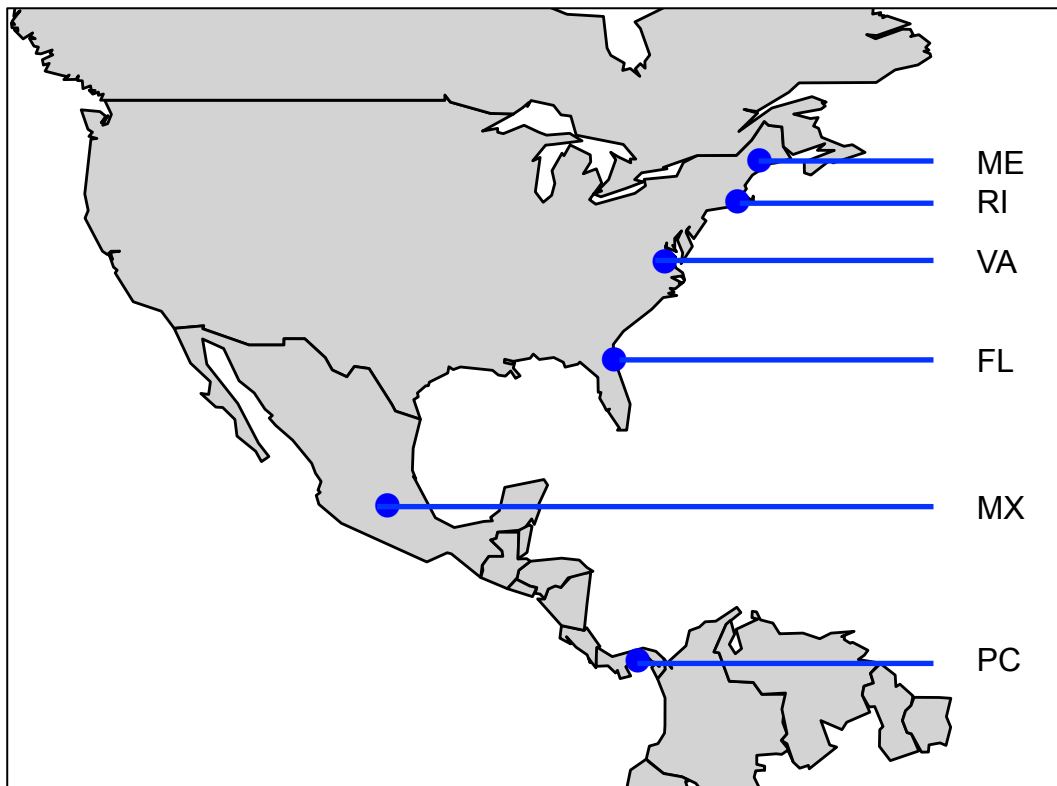

Supplement: S2 Fig — We studied a total of six D. melanogaster populations. Four of them originate from locations along the east coast of North America: ME in Fairfield, Maine (latitude: 44°37’N), RI in Providence, Rhode Island (41°49’N), VA in Richmond, Virginia (37°32’N), and FL in Jacksonville, Florida (30°20’N) (all sampled in September 2011). An additional population (PC) was sampled in Panama City, in Panama (8°58’N) in January 2012. A set of lines sampled from several locations in Mexico (mean latitude = 19°45’N) that were obtained from the Drosophila Species Stock Center at UCSD constituted our Mexico population sample (MX). (PDF) [file pgen.1005869.s002.pdf]

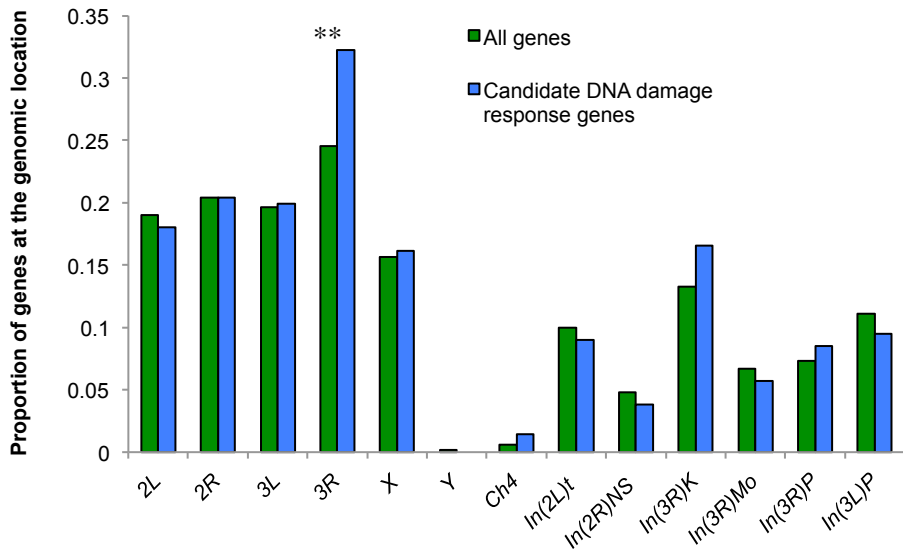

Supplement: S4 Fig — Information on physical location of candidate DNA damage response genes from FlyBase R6.04 was used to evaluate their genomic distribution. We found that with the exception of chromosome arm 3R (hypergeometric test: p = 0.006 (**); note: this p-value is significant after Bonferroni correction for multiple testing) none of the tested chromosome arms or regions (2L, 2R, 3L, X, Y, Chr4, In(2L)t, In(2R)NS, In(3R)K, In(3R)Mo, In(3R)P, In(3L)P) showed significant enrichment for UV damage response genes. This suggests that our candidate genes were slightly overrepresented on chromosome 3R, but not within the 3R inversions. (PDF) [file pgen.1005869.s004.pdf]

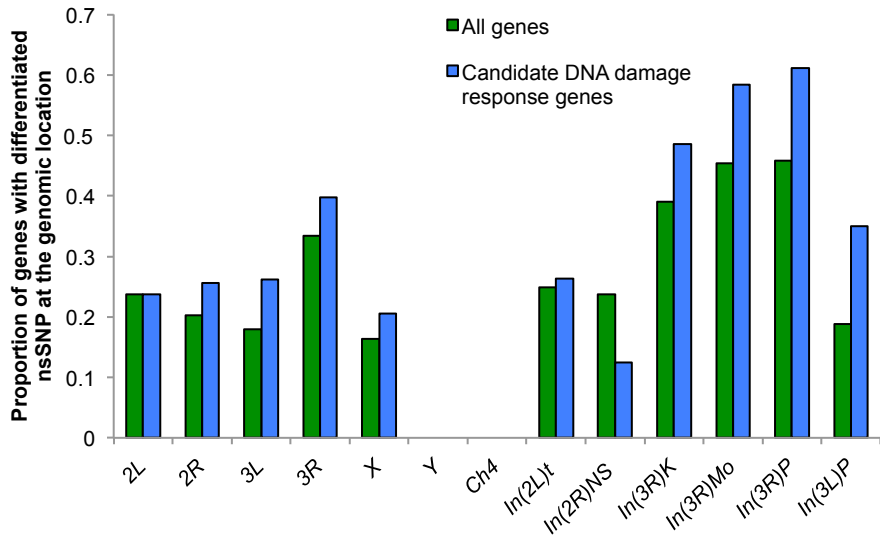

Supplement: S5 Fig — UV damage response genes with at least one differentiated nsSNP (FDR 0.001) were not significantly enriched in any of the tested chromosome arms or regions (2L, 2R, 3L, X, Y, Chr4, In(2L)t, In(2R)NS, In(3R)K, In(3R)Mo, In(3R)P, In(3L)P). This suggests that population differentiation in our candidate genes followed the general genomic pattern. (PDF) [file pgen.1005869.s005.pdf]

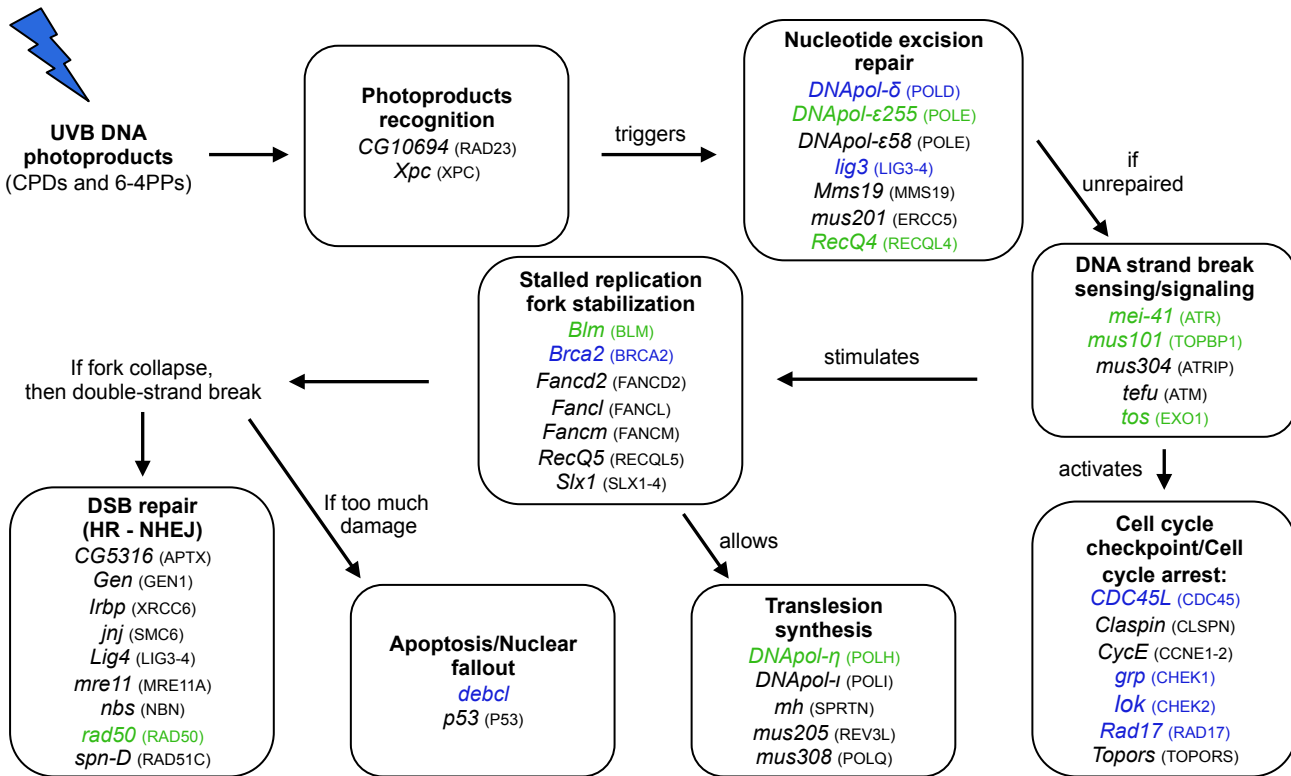

Supplement: S6 Fig — General pathways (bold black) and gene components (italic) and their human othologs (in brackets) showing significant differentiation between high and low latitudes. Genes that carry at least one differentiated non-synonymous polymorphism (FDR 0.001) are shown in black; genes with early embryo differential expression between high and low latitudes are shown in blue; genes that carried at least one differentiated nsSNP and were differentially expressed are shown in green. (PDF) [file pgen.1005869.s006.pdf]
