# Supplementary material for: The Adaptive Significance of Natural Genetic Variation in the DNA Damage Response of Drosophila melanogaster
Source: PLoS Genet. 2016 Mar 7;12(3):e1005869. doi: 10.1371/journal.pgen.1005869 (PMC4780809; doi:10.1371/journal.pgen.1005869)
Supplement: S4 Table — There were no discrepancies observed between the libraries or between the different gene classes that could generate spurious false positive differential expression patterns. (1 Fragments Per Kilobase of transcript per Million mapped reads) (PDF) [file pgen.1005869.s010.pdf]

Category	Central tendency	Panama (FPKM[1])	Rhode island (FPKM[1])
All expressed gene (FPKM[1] >1)	Median	29.54	28.73
	Mean	84.93	84.21
UV response genes	Median	45.12	41.57
	Mean	117.33	117.38
Differentially expressed UV response genes	Median	32.15	26.32
	Mean	82.94	82.69

[1] Fragments Per Kilobase of transcript per Million mapped reads
